# Examining the underlying processes of different dimensions of self-care behavior among persons with heart failure

**DOI:** 10.1186/s12872-022-02876-3

**Published:** 2022-10-06

**Authors:** Mohammed Munther Al-Hammouri, Jehad A. Rababah

**Affiliations:** Department of Community and Mental, Faculty of Nursing, Jordan University of Science and Technology, P.O. Box 3030, Irbid, 22110 Jordan

**Keywords:** Self-care, Heart failure, Impulsivity, Depression, Stress

## Abstract

**Purpose:**

This study was conducted to compare how potential predictors differentially contribute to the different dimensions of self-care in persons with heart failure.

**Methods:**

A cross-sectional design was used in this study. Purposive sampling was used to recruit a sample (N = 252) in this study. The data were collected from three major referral hospitals in Jordan. Data analysis was performed using multiple linear regression.

**Results:**

The results showed that around 89% of our sample had insufficiency in at least one dimension of self-care. The initial regression models showed that different combinations of predictors were significantly associated with different dimensions of self-care. These models were also different in terms of the variance explained and the coefficients of the significant predictors. After the follow-up analysis of the best fit models for these dimensions, these differences were maintained.

**Conclusion:**

Despite the similarities in the proposed variables predicting different self-care dimensions, their differences may suggest variations in the underlying processes controlling different self-care dimensions. The current study showed that seven out of the nine proposed variables predicted different dimensions of self-care either in the initial or best-fit models.

## Background

Self-care is "A naturalistic decision-making process" [1, p. 226]. Self-care is a group of different behaviors and attitudes that can be classified into three distinct dimensions (i.e., self-care maintenance, self-care confidence, and self-care management) [1]. Self-care maintenance is a group of behaviors that target the physiological integrity of the individual. Self-care management is the individual's ability to respond to the symptoms of heart failure when they occur. Self-care confidence is the individual's ability to perform self-care behaviors to slow disease progression. The literature has extensively examined the effect of self-care, represented by its three dimensions, on disease outcomes.

The three self-care processes were associated with slower disease progression and better health outcomes [2]. In addition, self-care behavior was associated with a better quality of life and fewer heart failure-related hospitalizations [3, 4]. There was also evidence for the association between heart failure and difficulty breathing sleep while sleeping if appropriate self-care was not adopted [5]. Besides, proper self-care minimizes repeated hospitalizations' economic and personal burdens [6]. Due to its importance, several studies have investigated the predictors of self-care.

Numerous studies examined predictors of self-care, including anxiety, depression, perceived control, educational level, health literacy, functional status, stress, heart failure knowledge, and impulsivity [7–11]. Despite the growing evidence on self-care predictors, further research is needed [7]. For example, among frequently studied variables, impulsivity was reported in only one study as a potential predictor of self-care [7]. Impulsivity is "an individualized, normative, and multidimensional pattern of human decision-making behavior characterized by free will and insufficient reasoning due to diminished reasoning process" [12, p. 7]. However, in the literature, impulsivity was examined concerning a single dimension of self-care (i.e., self-care maintenance) [7]. Psychological flexibility is another variable that is extensively studied in the literature and has the potential to predict human behavior, such as self-care.

Psychological flexibility consists of mental and psychological processes associated with various human and adaptive behaviors [13–15]. However, its association with self-care was not investigated yet. Impulsivity and psychological flexibility were originally derived from the acceptance and commitment therapy model, with preliminary evidence for predicting human behavior such as self-care in persons with heart failure [13]. Thus, the current study aimed to compare how potential predictors contribute to the different dimensions of self-care in persons with heart failure. This aim was accomplished using impulsivity, perceived stress, depressive symptoms, perceived control, heart failure knowledge, psychological flexibility, functional status, and demographic variables of age and time since diagnosis. Thus, the study will help replicate previous studies' results of the most frequently studied predictors concerning self-care dimensions. In addition, the study will help expand the previous studies' findings by introducing new potential predictors in this area (i.e., impulsivity and psychological flexibility).

## Methods

### Design and setting

This study was conducted using a cross-sectional design. Purposive sampling was used to recruit a sample to serve the goal of this study. The reason to use purposive sampling is that the self-care management scores can only be valid for those who have had heart failure symptoms in the last month. The data were collected from three major referral hospitals in Jordan.

### Sample

After obtaining approval from the ethics committee at Jordan University of Science and Technology, potential participants were invited to participate in the current study from a major referral hospital in Jordan. The only inclusion criterion for the present study was that the patient must have had trouble breathing or ankle swelling during the past month. The reason for setting this inclusion criterion is the scoring instructions for measuring the self-care management dimension.

The potential participants were approached by a trained research assistant and invited to the study. Those who agreed and met the inclusion criteria signed written informed consent after fully explaining the study. The participants completed paper-based questionnaires while they were visiting the hospital. The sample consisted of 252 participants who completed the study kit with a response rate of about 82%. The minimum sample size was estimated using G*Power software with the following parameters: alpha = 0.05, power = 0.8, small to medium effect size (0.07), and nine predictors [16]. The minimum sample size required was 223.

### Data collection

We collected the data using a demographic questionnaire (developed for this study), the Self-Care of Heart Failure Index (Version 6.2), Barrett Impulsiveness Scale (BIS-11), Patient Health Questionnaire–9 (PHQ-9), Perceived Stress Scale-10 (PSS-10), Control Attitudes Scale-Revised (CAS-R), New York Heart Association (NYHA) functional classification, Atlanta Heart Failure Knowledge Test Version 3 (AHFKT), and Acceptance and Action Questionnaire (AAQ 2) to measure the study variables.

#### Outcome variables

Self-care maintenance, self-care management, and self-care confidence (i.e., self-care dimensions) were measured using the Self-Care of Heart Failure Index, Version 6.2 (SCHFI-V6.2) [17]. Scores for all three scales were standardized by converting each subscale score to a 100-point scale [17]. Higher scores indicate better self-care practices in all dimensions: maintenance, management, and confidence. The cutoff point for sufficient self-care processes using the SCHFI-V6.2 is 70 [17]. The Cronbach's alpha for the three scales ranged from 0.61 to 0.72 in persons with heart failure [7].

#### Potential predictors

The impulsivity of the study participants was evaluated using the BIS-11. The BIS-11 is a commonly used method to measure impulsivity [18]. It has 30 items with four response options from one "Rarely or Never" to four "Almost Always." The total BIS-11 score was calculated by summing up the scores of individual items after reverse-scoring negatively worded items. The Cronbach's alpha of the BIS-11 ranged from 0.71 to 0.83 [18]

Depressive symptoms were assessed using the PHQ-9, consisting of nine items [19]. These items ask about problems faced by the participants in the last two weeks, with higher scores indicating higher levels of depressive symptoms (i.e., a score of 5 = mild depression, a score of 10 = Moderate depression, a score of 15 = moderate-to-severe depression, and a score of 20 = severe depression). The Cronbach's alpha for the PHQ was 0.83 [19].

Perceived stress was measured using the PSS-10, with ten items relevant to the perceived stress of the participant [20]. A higher PSS-10 score indicates higher perceived stress. The Cronbach's alpha for the PSS-10 was 0.75 [7]. Perceived control was assessed using the CAS-R [21]. It consists of eight items with a 5-point-Likert-like scale ranging from one (i.e., totally disagree) to five (i.e., totally agree), with higher total scores indicating higher perceived control. The Cronbach's alpha for the CAS-R was greater than 0.70 [21].

Functional status was assessed using the NYHA [22]. The NYHA was first created in 1963 [23]. The NYHA class ranges from one (i.e., no symptoms with ordinary physical activity) to four (i.e., symptoms occur at rest). The NYHA asks about the severity of the heart failure symptoms concerning the level of activities being performed [22]. The trained research assistant administered the NYHA.

Heart failure knowledge was assessed using the AHFKT. The AHFKT has 30 questions about heart failure knowledge: Nutrition, heart failure symptoms, behavior, medications, and pathophysiology [24]. The total score represents the number of correctly answered questions, with higher total scores indicating better heart failure knowledge. The Cronbach's alpha for AHFKT was 0.87 [24].

Psychological flexibility was assessed using the AAQ-II [25]. The AAQ-II is seven items, single-factor measure used to evaluate psychological flexibility. It uses a 7-point Likert-like response scale from one (i.e., never true) to seven (i.e., always true). The Cronbach's alpha for the AAQ-II was 0.84 [25].

#### Data analysis

The data analysis was performed using SPSS (v. 23). Preliminary analysis was performed (i.e., descriptive statistics and frequencies). In the preliminary analysis, the following linear regression assumptions were tested: Normality, homoscedasticity, linearity, and multicollinearity. During this step, no issues were detected, and the authors moved forward with the linear regression analysis and interpretation of the results. The self-care dimensions were measured using SCHFI V6.2, which provides three different scores for each scale (i.e., the outcome variables). Multiple linear regression was performed to compare the three different dimensions of self-care.

## Results

Initially, we ran descriptive statistics of the study sample (see Table 1). Then, we ran descriptive statistics on our study variables (see Table 1). The mean average age was 62 years (*SD* = 13.45), with 72.6% males and 27.4% females. Tables 1 and 2 summarize participants' characteristics. In our current sample, about 80% of the sample scored below 70 on health care maintenance, about 67.3% scored below 70 on self-care confidence, and about 69.1% scored below 70 on self-care management. To make more sense of these results, we closely examined the trend of lacking sufficient self-care in all three scales. We found that 49.2% (*n* = 124) have insufficient self-care on all of the three self-care dimensions (i.e., scored below 70 in all of the three self-care dimensions), 28.8% (*n* = 72) have insufficient self-care in two self-care dimensions (i.e., scored below 70 in two of the self-care dimensions), 11% (*n* = 28) have insufficient self-care on only one self-care dimension (i.e., scored below 70 in one of the three self-care dimensions), and only 11% (*n* = 28) have sufficient self-care in all three dimensions (i.e., scored over 70 in all of the three self-care dimensions).

**Table 1:** Sample characteristics (*N* = 252)

Variable	*M (SD)*
Age (years)	62.00 (13.45)
Time since diagnosis (months)	22.62 (38.39)
Self-care maintenance	52.97 (19.68)
Self-care confidence	61.41(20.60)
Self-care management	54.90 (21.05)

	n (%)
*Functional status*	
I	38 (15.0)
II	87 (34.5)
III	83 (32.9)
IV	44 (17.6)
*Gender*	
Female	69 (27.4)
Male	183 (72.6)
*Marital status*	
Married	195 (77.4)
Widow	40 (15.9)
Divorced	17 (6.7)
*Employment*	
Employed	183 (72.6)
Retired	69 (27.4)
*Highest education*	
High school	115 (45.6)
Diploma	84 (33.8)
Bachelor's	52 (20.6)

*N* Sample size, *SD* Standard deviation, *M* Mean, *n* number of participants

**Table 2:** Linear regression results of different self-care dimensions

	Self-care maintenance				Self-care management				Self-care confidence			
Model summary	R^2^	F	df1, df2	*p*	R^2^	F	df1, df2	*p*	R^2^	F	df1, df2	*p*
	.46	23.02	9, 242	.000	.23	8.45	9, 242	.000	.26	9.69	9, 242	.000

Predictor	B	SE	*β*	*p*	B	SE	*β*	*p*	B	SE	*β*	*p*
Constant	30.88	12.02		**.011**	24.92	15.37		.106	33.13	14.75		**.026**
Impulsivity	− .309	.109	− .16	**.005**	− .29	.14	− .13	**.041**	− .36	.13	− .17	**.007**
Stress	− .45	.19	− .15	**.023**	− .18	.25	− .06	.471	− .47	.24	− .15	**.049**
Depression	− .25	.18	− .09	.181	.35	.23	.12	.141	.08	.22	.03	.705
Perceived control	.49	.20	.13	**.015**	.48	.25	.11	.061	1.39	.24	.35	**.000**
HF knowledge	1.31	.16	.42	**.000**	1.37	.21	.40	**.000**	.41	.20	.13	**.040**
Age	.22	.07	.15	**.003**	.11	.10	.07	.241	.09	.09	.05	.351
Time since diagnosis	.01	.02	.02	.658	.03	.03	.05	.392	.01	.03	.02	.710
Psychological flexibility	.14	.12	.07	.266	.23	.16	.11	.152	.37	.15	.17	.**016**
Functional status	− .71	.89	− .04	.426	− .92	1.15	− .05	.421	− .13	1.10	− .01	.906

*R*^2^ Explained variance, *F* F-statistic, *df* Degrees of freedom, *p* p-value, *B* Regression coefficient, *SE* Standard error, *β* Standardized coefficient, *t*: *HF* Heart failure. Bold: *p* value < .05

We compared the three self-care dimensions in persons with heart failure by regressing self-care maintenance, self-care management, and self-care confidence on the potential predictors (i.e., impulsivity, perceived stress, depressive symptoms, perceived control, heart failure knowledge, psychological flexibility, functional status, age, and time since diagnosis). Table 2 summarizes the results of the multiple linear regression. For self-care maintenance, the regression model was statistically significant [*F* (9, 242) = 23.02, *p* < 0.001]. The R^2^ was 0.46, meaning that the model explained 46% of the variance in self-care maintenance. Among the predictor variables entered into the model, impulsivity, perceived stress, perceived control, heart failure knowledge, and age were the significant predictors (Table 2).

Regarding self-care management, the tested model was also significant [*F* (9, 242) = 8.45, *p* < 0.001]. The R^2^ was 0.23, meaning the variance explained in self-care management was 23%. Only two predictors were significant: impulsivity and heart failure knowledge (Table 2). For self-care confidence, the results showed that the model was significant [*F* (9, 242) = 9.69, *p* < 0.001]. The R^2^ for the model was 0.26, meaning that the model explained 26% of the self-care confidence variance. The significant predictors in the model were impulsivity, heart failure knowledge, psychological flexibility, perceived stress, and perceived control.

A follow-up analysis was performed to find the best fit model for each self-care dimension. This was done to check the model fitness while including the significant predictors and excluding the nonsignificant ones, as identified in the previous step. Table 3 summarizes the best fit model results. The results are consistent with the initial regression results presented in Table 2 regarding the significance of the models, explained variance, and the number of significant predictors in the model, even after excluding nonsignificant predictors from the models. The only difference was in the number of significant predictors for the self-care management dimension. In the initial model (Table 2), impulsivity and heart failure knowledge were the only significant predictors of self-care management. On the other hand, the best fit model (Table 3) shows four significant predictors of self-care management: Impulsivity, heart failure knowledge, depressive symptoms, and perceived control. For self-care management, this means that the excluded predictors masked the effect of depressive symptoms and perceived control in the initial regression model.

**Table 3:** Best-fit models for self-care dimensions

	Self-care maintenance			
Model summary	R^2^	F	df1, df2	*p*
	.46	41.00	5, 246	.000

Predictor	B	SE	*β*	*p*
Constant	28.71	11.47		.013
Age	0.21	0.07	0.14	.004
Impulsivity	− 0.31	0.11	− 0.15	.005
Stress	− 0.49	0.15	− 0.17	.002
Perceived control	0.53	0.19	0.14	.007
HF knowledge	1.34	0.16	0.42	.000

	Self-care management			
Model summary	R^2^	F	df1, df2	*p*
	.22	17.91	4, 247	.000

Predictor	B	SE	*β*	*p*
Constant	27.77	13.15		0.03
Impulsivity	− 0.27	0.14	− 0.13	0.04
Depression	0.40	0.19	0.14	0.03
Perceived control	0.54	0.25	0.13	0.03
HF knowledge	1.37	0.20	0.40	.000

	Self-care confidence			
Model Summary	R^2^	F	df1, df2	*p*
	.26	17.34	5, 246	.000

Predictor	B	SE	*β*	*p*
Constant	38.20	12.75		.003
Impulsivity	− 0.35	0.13	− 0.17	.008
Stress	− 0.44	0.21	− 0.14	.041
Perceived control	1.39	0.24	0.35	.000
Psychological flexibility	0.36	0.14	0.17	.013
HF knowledge	0.41	0.19	0.12	.032

*R*^2^ Explained variance, *F* F-statistic, *df* Degrees of freedom, *p* p-value, *B* Regression coefficient, *SE* Standard error, ***β*** Standardized coefficient, *t*: *HF* Heart failure

## Discussion

Self-care in persons with heart failure is a multidimensional behavior that involves three distinct dimensions: self-care maintenance, self-care confidence, and self-care management. However, little is known about the difference in the predicting variables of these dimensions. This study aimed to compare how potential predictors contribute to the different dimensions of self-care. The current study results are consistent with the literature. For example, impulsivity is a significant predictor of the self-care maintenance dimension [7]. However, the current study expanded the previous work by showing a similar effect of impulsivity on other self-care dimensions.

Interestingly, depressive symptoms were not a significant predictor of self-care for the three self-care dimensions in the initial model (Table 2). However, depressive symptoms were a significant predictor of the self-care management dimension in the follow-up analysis of the best models of self-care dimensions (Table 3). These results were consistent with recent studies incorporating impulsivity in regression models to predict self-care [7, 11]. In addition, the results of the current study were consistent with the previous research findings on the importance of heart failure knowledge, perceived stress, and perceived control in predicting self-care [7–11].

Categorizing self-care into sufficient and insufficient self-care in the three dimensions based on the standardized scores of the self-care measure helped us understand the magnitude of self-care problems. For example, only 11 percent of our sample showed sufficient self-care in all three dimensions. In other words, about 90 percent of our participants showed insufficient self-care at least in one dimension, which is consistent with previous research [7, 17, 26].

A careful look at Table 2 provides several insights. Firstly, impulsivity and heart failure knowledge are the only two significant predictors across the three dimensions of self-care. Secondly, different dimensions of self-care had different combinations of predictors. For example, where five predictors significantly predicted self-care maintenance, only two of them predicted self-care management (Table 2). Thirdly, the standardized coefficients indicated that variation exists in terms of the individual effect of each predictor on different dimensions of self-care. For example, the heart failure knowledge standardized coefficients were 0.42 for self-care maintenance, 0.40 for self-care management, and 0.13 for self-care confidence. On the other hand, impulsivity was the only predictor that showed a stable effect on the three self-care dimensions (Table 2).

The best fit model analysis supported the initial analysis except for self-care management, where depressive symptoms and perceived control became significant predictors in the model. In addition, seven of the nine proposed predictors in this study showed statistical significance in predicting at least one self-care dimension, either in the initial or best-fit models. These predictors are impulsivity, perceived stress, depressive symptoms, perceived control, heart failure knowledge, age, and psychological flexibility. This finding signifies the complex nature of self-care behavior in persons with heart failure.

Regarding impulsivity and psychological flexibility, the literature about their association with self-care behavior in persons with heart failure is minimal, limiting comparing the current study results with previous research findings. However, considering the evidence on other behaviors in different populations, the direction of the association between these variables and self-care dimensions is consistent with previous studies on other behaviors in diverse populations [7, 13]. It is worth noting that impulsivity has predicted all dimensions of self-care behavior in persons with heart failure. At the same time, psychological flexibility was a significant predictor of only Self-care Confidence. This warrant further research in this area to better understand the role of these variables on self-care behavior dimensions in persons with heart failure.

The current study showed that heart failure knowledge, perceived stress, and perceived control were significant predictors of all self-care behavior dimensions. These results were consistent with previous research findings [7–11, 27]. On the other hand, depression symptoms were only a predictor of self-care management, but not self-care confidence and maintenance. These results were inconsistent with some previous research findings [28]. However, some other evidence is consistent with our study findings [7]. Depressive symptoms have been previously claimed as a significant predictor of different aspects of self-care in persons with heart failure. The results reported here showed that depressive symptoms are only responsible for predicting self-care management, which is consistent with other research studies. On the other hand, the evidence regarding the role of impulsivity has not gained attention in self-care among persons with heart failure until recently. The results of this study revealed that impulsivity is a significant predictor of all three aspects of self-care in persons with heart failure. In other words, the role of impulsivity could be more important clinically than the role of depressive symptoms.

The implications of the current study results extend to the conceptual understanding of self-care. Despite the similarities in different models of self-care dimensions, the differences suggest that self-care dimensions might be controlled, at least partially, by different underlying processes. However, this conclusion needs further research to be supported. These results lead us to another possible implication: incorporating newly suggested predictors into future self-care improvement research and interventions. Manipulating a person's environment to promote adaptive and healthy behavior requires collaboration between healthcare professionals, heart failure persons, and caregivers. Thus, intervention training may also include the caregivers of persons with chronic illnesses, requiring further investigation if non-professionals, such as family caregivers, apply these interventions.

## Limitations

The study results are limited by the sampling approach used. Besides, the ratio of males to females should be considered while reading the current study results. Additionally, the study sample represented Middle eastern persons with heart failure. Therefore, we believe that there is a need to replicate this study in different settings, cultures, and ethnic groups. In addition, the current study did not collect any data about biological indicators of health or quality of life that may give further insight into self-care dimensions. Although self-care confidence is not a behavioral component of self-care, it still can be considered an essential factor in determining self-care in persons with heart failure.

## Conclusion

The current study showed that self-care is sub-optimal in persons with heart failure, and it also replicated newly proposed predictors or expanded on the newly proposed ones. Thus, the result could guide future research in modifying, applying, and examining interventions to promote self-care in heart failure persons. However, future research must answer questions regarding the effectiveness of such interventions in persons with heart failure.

## Data Availability

The datasets used and/or analyzed during the current study are available from the corresponding author on reasonable request.

## Abbreviations

AHFKT: The Atlanta heart failure knowledge test
BIS-11: Barret impulsiveness scale
CAS-R: The control attitudes scale-revised
F: F-test
NYHA: New York heart association classification
*P*: *P* Value
PHQ-9: The patient health questionnaire–9
AAQ-II: The acceptance and action questionnaire
R^2^: R-squared
SCHFI-V6: The self-care of heart failure index version 6.2
SD: Standard deviation
